# Perceived oral health in patients after bariatric surgery using oral health‐related quality of life measures

**DOI:** 10.1002/cre2.134

**Published:** 2018-10-16

**Authors:** Lena Karlsson, Johanna Carlsson, Kristina Jenneborg, Marianne Kjaeldgaard

**Affiliations:** ^1^ Section of Cariology and Endodontics, Division of Oral Diseases, Department of Dental Medicine Karolinska Institutet Sweden

**Keywords:** bariatric surgery, oral health, oral health‐related quality of life, patient‐reported outcome measures

## Abstract

Obesity is an increasing problem of the 21st century. A frequent intervention is bariatric surgery. The impact of bariatric surgery on oral health is largely unknown. The aim of the present case–control study was to survey the perceived oral health amongst individuals that had undergone bariatric surgery and compare the measures with two cohorts consisting of healthy individuals with respectively at or below versus above a body mass index score of 30. Study volunteers were recruited from interest group on the Internet. The study participants completed online a validated oral health‐related quality of life scale, that is, OHIP‐S. The three cohorts consisted of individuals that had undergone bariatric surgery (OS, *n* = 77) and the healthy obese (ONS, *n* = 45) and nonobese individuals (HNS, *n* = 71). Nonparametric Kruskal–Wallis rank sum tests were used to estimate likelihood of nondifference amongst the three cohorts. Individuals that had undergone bariatric surgery reported significant more oral health problems than the study participants in with the two other cohorts. Their perception of oral health‐related quality of life was higher or similar to the obese study participants and lower than for nonobese study participants. Perceived oral health problems appear to be more frequent amongst individuals that have undergone bariatric surgery in comparison with healthy obese and nonobese individuals. Bariatric surgery may be consider a risk marker for impaired oral health.

## 1 BACKGROUND

Obesity is an increasing problem of the 21st century and one of the fastest growing health problems worldwide (Buchwald & Williams, 2004; Flodgren, Goncalves‐Bradley, & Summerbell, 2017). Obesity may contribute to the occurrence of various health conditions such as diabetes mellitus, cardiovascular disease, stroke, hypertension, and certain cancer forms (Marsicano, Sales‐Peres, Ceneviva, & de Sales‐Peres, 2012; Vairavamurthy, Cheskin, Kraitchman, Arepally, & Weiss, 2017; Vinciguerra et al., 2013) and associates with oral health problems such as dental caries, periodontitis, and hyposalivation (Marsicano, Sales‐Peres, Ceneviva, & de Sales‐Peres, 2012). Bariatric surgery is considered to be one of the most effective and a safe method to achieve weight loss in adults and may improve systemic conditions (Barbosa et al., 2009; Costa, Yamaguchi, Santo, Riccioppo, & Pinto‐Junior, 2014). However, bariatric surgery may also have negative impact on oral health conditions because of an increase in periodontal disease, caries, and dental wear (de Moura‐Grec et al., 2014; Heling et al., 2006). One potential side effect of bariatric surgery is gastroesophageal reflux disease (Basoglu et al., 2015). Recurrent high acidic level in the oral cavity is a well‐known risk factor associated with dental caries, tooth erosion, and dental hypersensitivity (Barbosa et al., 2009; Watanabe et al., 2017; West et al., 2013). The diet consumption pattern following bariatric surgery, which may include increasing intake frequency of food and drinks, is also a well‐known potential risk factor for developing dental caries (Jastrzebska‐Mierzynska, Ostrowska, Wasiluk, & Konarzewska‐Duchnowska, 2015; Mechanick et al., 2013). Binge eating disorder (Dawes et al., 2016) and vomiting are other reported side effects (Barbosa et al., 2009), which can lead to a variety of potential dental problems. Also periodontal disease seems to be more common in individuals that have undergone bariatric surgery and may be related to nutritional deficiency secondary to the surgery (de Moura‐Grec et al., 2014; Netto et al., 2012). The material available about oral health and postbariatric surgery is limited and sometimes contradictory. Pataro et al. (2012) investigated the prevalence of periodontitis in individuals that have undergone bariatric surgery before and after surgery and found no postoperative significant difference. However, Cummings and Pratt (2015) stated in their study that periodontal disease increases after bariatric surgery. Sales‐Peres et al. (2015) evaluated if periodontal disease and periopathogens were influenced by gastric bypass operation, and their findings showed an increased periodontal disease in the study group. This was in accordance with the findings of Marsicano et al. (2012) and Netto et al. (2012), where a small difference in increased dental pocket depth in individuals that have undergone bariatric surgery was demonstrated, compared with obese patients who had not undergone bariatric surgery. Netto et al. (2012) also reported that vitamin C deficiency and increased vomiting after surgery might be an explanation to increased periodontal disease postoperative in the study group. A cohort study (de Moura‐Grec et al., 2014) evaluated oral health conditions before and 6 months after bariatric surgery, and the results showed that systemic conditions could improve but oral health conditions deteriorated because of increased periodontal disease and decayed teeth. To summarize, the majority of the limited amount of published studies in the field shows that different postoperative side effects associated with bariatric surgery express common risk factors for several oral health problems. Dietary intake pattern, vomiting, gastroesophageal reflux disease, and nutritional deficiency can all be risk factors for increased dental wear, prevalent dental hypersensitivity, higher frequency of caries lesions, and periodontal disease (Barbosa et al., 2009; de Moura‐Grec et al., 2014; Jastrzebska‐Mierzynska et al., 2015; Mechanick et al., 2013; Netto et al., 2012; Watanabe et al., 2017; West et al., 2013). Because the number of bariatric surgeries has increased extensively in recent years (Bouldin et al., 2006), a question has been raised about whether changes in oral health condition after the significant weight loss or unsatisfactory oral health conditions before bariatric surgery might worsen after surgery. The aim of the present case–control study was to survey the perceived oral health amongst individuals that had undergone bariatric surgery and compare the measures with two cohorts consisting of healthy individuals with a body mass index (BMI) score respectively below and above 30. Our hypothesis was that self‐reported oral health was negatively impacted following bariatric surgery.

## 2 MATERIAL AND METHODS

### 2.1 Study group

**Figure 1:**
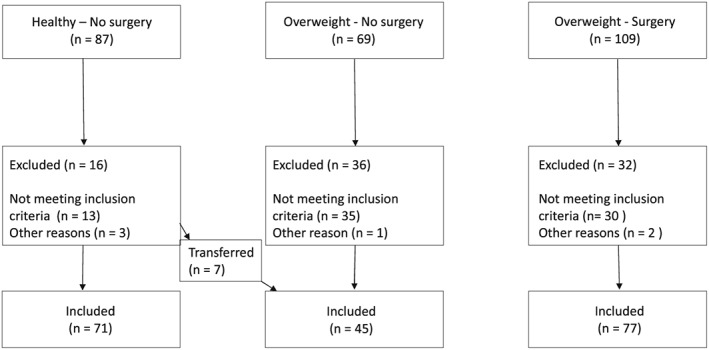
Flow chart. Totally, 193 informants were included and distributed—after scrutinizing the inclusion and exclusion criteria—in group healthy non‐obese patients, in group overweight—no surgery group, or in group overweight—surgery group

Two hundred sixty‐five participants were invited to take part in the study: 109 in the OS group, 69 in the ONS group, and 87 in the HNS group. The study participants were recruited from closed groups on the Internet consisting of individuals who had undergone bariatric surgery or considering the intervention. Those who had undergone surgery in the past year were excluded because it was assumed that the operation would not have affected oral health until after the first year following bariatric surgery. Some of the study participants that at the time were considering undergoing bariatric surgery were also asked to participate in the study when attending an information event about bariatric surgery at a hospital, Sophiahemmet, in Sweden. The HNS study participants were recruited from the Internet through a public request to answer the survey. The criterion for participation in (a) the OS group was to have undergone bariatric surgery, (b) the ONS group to have a BMI ≥30, and (c) the HNS group to have a BMI <30. Following the completion of the survey (Figure 1), study participants from the HNS group (*n* = 7) were transferred to the ONS group due to declaring a higher BMI than 30. Six study participants from the HNS group were excluded due to missing data about BMI. One study participant was excluded due to declaring a high BMI because of pregnancy, and two study participants were excluded due to duplication of the answers. In the ONS group, 25 study participants were removed due to declaring they had undergone bariatric surgery, five study participants were excluded due to missing data about BMI, and one study participant was excluded due to obvious duplication of the answers. In the OS group, 11 study participants were excluded due to missing data about BMI, and two study participants were excluded due to duplication of the answers. Seventeen study participants in the OS group were also excluded due to undergone surgery during the study period, and two study participants were excluded due to missing date/year of surgery. The study was approved by the Regionala Etikprövningsnämnden (EPN), Ethics Committee in Stockholm, Sweden (2008/1734‐31/3). The declaration of World Medical Association of Helsinki, as well as human rights, has been taken into consideration throughout the study.

### 2.2 Study design

Oral health‐related quality of life was determined by means of a validated self‐reported oral health measure system in Swedish (OHIP‐S; Larsson, List, Lundstrom, Marcusson, & Ohrbach, 2004). Some of the questions were modified in order to suit the purpose of the study. The questionnaire (Table 1) was web‐based, anonymous, and all responders (*n* = 193) were asked to answer 31 closed questions. The head formulation for all questions was “How often have you during the last year …:” The different response categories were represented as *all the time*, *very often*, *fairly often*, *sometimes*, *seldom*, *never*, and *N/A*. The collected data included descriptive data about the patient as well as the result of the OHIP‐S questionnaire with multiple‐choice questions. The questions included date of birth, occupation, BMI (?), and weight. The participants who had undergone bariatric surgery were asked to provide the BMI before surgery and after surgery. Medical (nondental) questions included a general feeling of well‐being, dietary intake, vomiting, indigestion, heartburn, number of meals per day, and consumption of sweet foods. Oral health questions addressed the participants' perceived oral health and included oral hygiene practices, dental hypersensitivity, and dental wear.

**Table 1:** The 31 questions from the OHIP‐S included in the present online survey

1	Noticed a tooth that does not look like it should?
2	Difficulties with chewing any kind of food due to dental problems?
3	Have you had problems with dental caries?
4	Experienced mouth dryness?
5	Have you felt that you have had bad breath?
6	Experienced that your ability to feel taste has deteriorated?
7	Experienced tooth sensitivity while consuming hot or cold food or drink?
8	Experienced toothache?
9	Experienced pain in the gums, oral mucosa, or tongue?
10	Experienced problems with bleeding gums?
11	Brushed your teeth at least 2 times a day?
12	Used fluoride toothpaste when brushing your teeth?
13	Used dental floss, toothpick, or interdental toothbrush more than 3 times a week?
14	Used any kind of fluoride supplements in addition to fluoride toothpaste, such as fluoride gum, fluoride tablets, or fluoride mouth rinse?
15	Consumed food, snacks, or drinks (water not included) more than 5 times a day?
16	Have you been forced to avoid eating some types of food?
17	Have you consumed sweets or sweet drinks once a day or more often?
18	Experienced problems with acid reflux?
19	Experienced problems with vomiting?
20	Have you felt anxious due to dental problems?
21	Have you felt bothered by your appearance?
22	Have you felt bothered by your appearance because of your teeth?
23	Have you been avoiding to smile?
24	Experienced that life has generally been less satisfactory?
25	Have you felt low or depressed?
26	Have you felt that your current well‐being has deteriorated?
27	Have you been unable to appreciate other people's company?
28	Experienced insomnia?
29	Experienced difficulties with your ability to concentrate?
30	Experienced tiredness or lack of energy?
31	Experienced difficulties in managing everyday tasks?

### 2.3 Statistical analysis

All data were analyzed using R Core Team software (version 3.3.3.2017, Vienna, Austria). The statistical methods used were the Likert scale and Kruskal–Wallis rank sum test. The Likert scale is a psychometric scale often used in studies where questionnaires are applied. Kruskal–Wallis rank sum test is a nonparametric test often used for comparing two or more independent samples (or groups) of equal or different sample sizes. The significance level was set at a 1% level and a 5% level (*P* < 0.01 and <0.05). The Likert scaling presents responses for each question and was divided into three categories: low—negative response (as in responses as *no* and *never*), neutral—neutral response (as in responses as *don't know* and *sometimes*), and high—positive response (as in responses as *yes* and *very often*).

## 3 RESULTS

Table 2 displays the descriptive statistics for each group regarding age, BMI, and gender. The sample group of overweight surgery patients (OS) consisted of 77 patients (mean BMI = 27.78): 76 females (98.70%) and 1 male (1.30%) with a mean age of 42.88 years. The first control group consisting of overweight no surgery (ONS) study participants comprised 45 study participants (BMI = 38.96): 42 females (93.3%) and 3 males (6.67%) with a mean age of 43.83 years. The second control group consisting of individuals with normal weight and no surgery (HNS) comprised 71 study participants (BMI = 24.01): 57 females (80.28%) and 14 males (19.72%) with a mean age of 34.49 years. Findings from the survey are presented in Table 3. The results from the Likert analysis are divided into three categories and are represented in the left column, whereas the results from the Kruskal–Wallis rank sum test are depicted in the right column. The Likert scaling presents the proportion of the responses (in %) within each category for every group, as well as mean value and standard deviation of the ordinal scale for each group. In order to calculate the arithmetic mean, ranks from 1 to 3 or from 1 to 5, depending on the number of choices for each question, have been assigned to each response according to the ordinal scale, from negative to positive response. In the Kruskal–Wallis part are three columns presented, statistic, *P* value, and significance code: “**”: *P* value <0.01 and “*”: *P* value <0.05. The following questions showed a statistically significant difference for one or two obese groups (OS and ONS; *P* < 0.01, *P* < 0.05).

**Table 2:** Descriptive statistics for each group (HNS, ONS, and OS) included in the study regarding age, BMI, and gender

Group	Variable	Levels	*N*	Mean	*SD*	Min	Max	%
Healthy—No surgery	Age		71	34.49	10.08	19.00	69.00	
Overweight—No surgery			45	43.82	10.48	24.00	70.00	
Overweight—Surgery			77	42.88	10.79	22.00	71.00	
Healthy—No surgery	BMI		71	24.01	2.56	18.50	29.80	
Overweight—No surgery			45	38.96	5.91	30.10	56.50	
Overweight—Surgery			77	27.78	4.09	17.80	40.40	
Healthy—No surgery	*ΔBMI*		—	—	—	—	—	
Overweight—No surgery	‐ post op		—	—	—	—	—	
Overweight—Surgery			77	−14.94	4.50	−29.20	−6.90	
Healthy—No surgery	Gender	Female	57					80.28
		Male	14					19.72
Overweight—No surgery		Female	42					93.33
		Male	3					6.67
Overweight—Surgery		Female	76					98.70
		Male	1					1.30

*Note*. BMI: body mass index.

**Table 3:** Results analyzed from the questionnaire

	Healthy—No surgery	Overweight—No surgery	Overweight—Surgery	Statistic	*P* value	Significance
Question	1	1	1	1.18	0.55	
Low^a^	22.54%	33.33%	35.06%			
Neutral^b^	77.46%	60.00%	59.74%			
High^c^	0.00%	6.67%	5.19%			
Mean	1.77	1.73	1.70			
*SD*	0.42	0.58	0.56			
Question	2	2	2	10.95	<0.01	**
Low	67.61%	55.56%	51.95%			
Neutral	29.58%	31.11%	15.58%			
High	2.82%	13.33%	32.47%			
Mean	2.14	2.69	3.31			
*SD*	1.52	1.99	2.24			
Question	3	3	3	9.53	<0.01	**
Low	46.48%	37.78%	35.06%			
Neutral	33.80%	33.33%	22.08%			
High	19.72%	28.89%	42.86%			
Mean	3.15	3.87	4.22			
*SD*	1.92	1.89	2.16			
Question	4	4	4	5.88	0.05	
Low	38.03%	48.89%	33.77%			
Neutral	38.03%	24.44%	24.68%			
High	23.94%	26.67%	41.56%			
Mean	3.41	3.47	4.21			
*SD*	1.99	2.16	2.04			
Question	5	5	5	0.65	0.72	
Low	56.34%	53.33%	53.25%			
Neutral	1.41%	0.00%	3.90%			
High	42.25%	46.67%	42.86%			
Mean	3.76	3.71	3.56			
*SD*	1.28	1.65	1.65			
Question	6	6	6	35.86	<0.01	**
Low	95.77%	82.22%	61.04%			
Neutral	0.00%	4.44%	3.90%			
High	4.23%	13.33%	35.06%			
Mean	1.66	2.16	3.22			
*SD*	1.09	1.58	1.72			
Question	7	7	7	8.3	0.02	*
Low	78.87%	55.56%	58.44%			
Neutral	0.00%	2.22%	7.79%			
High	21.13%	42.22%	33.77%			
Mean	2.75	3.47	3.45			
*SD*	1.50	1.65	1.64			
Question	8	8	8	14.15	<0.01	**
Low	56.34%	42.22%	38.96%			
Neutral	35.21%	44.44%	25.97%			
High	8.45%	13.33%	35.06%			
Mean	2.39	2.98	3.44			
*SD*	1.62	1.67	1.76			
Question	9	9	9	6.5	0.04	*
Low	85.92%	62.22%	70.13%			
Neutral	0.00%	2.22%	1.30%			
High	14.08%	35.56%	28.57%			
Mean	2.42	3.27	2.95			
*SD*	1.43	1.71	1.86			
Question	10	10	10	3.09	0.21	
Low	32.39%	35.56%	33.77%			
Neutral	45.07%	20.00%	25.97%			
High	22.54%	44.44%	40.26%			
Mean	3.56	4.09	4.06			
*SD*	1.83	2.12	2.06			
Question	11	11	11	0.78	0.68	
Low	78.87%	82.22%	75.32%			
Neutral	1.41%	4.44%	9.09%			
High	19.72%	13.33%	15.58%			
Mean	3.18	2.98	3.12			
*SD*	1.10	1.12	1.12			
Question	12	12	12	1.7	0.43	
Low	5.63%	11.11%	1.30%			
Neutral	87.32%	75.56%	88.31%			
High	7.04%	13.33%	10.39%			
Mean	3.97	3.98	4.16			
*SD*	0.76	0.92	0.54			
Question	13	13	13	1.34	0.51	
Low	50.70%	42.22%	45.45%			
Neutral	19.72%	26.67%	20.78%			
High	29.58%	31.11%	33.77%			
Mean	3.49	3.82	3.73			
*SD*	1.67	1.37	1.56			
Question	14	14	14	0.98	0.61	
Low	61.97%	57.78%	49.35%			
Neutral	12.68%	6.67%	14.29%			
High	25.35%	35.56%	36.36%			
Mean	3.17	3.16	3.40			
*SD*	1.59	1.80	1.69			
Question	15	15	15	4.21	0.12	
Low	32.39%	37.78%	10.39%			
Neutral	14.08%	20.00%	63.64%			
High	53.52%	42.22%	25.97%			
Mean	4.42	3.82	4.22			
*SD*	1.55	1.60	1.05			
Question	16	16	16	31.3	<0.01	**
Low	66.20%	60.00%	33.77%			
Neutral	16.90%	22.22%	18.18%			
High	16.90%	17.78%	48.05%			
Mean	2.44	2.69	4.34			
*SD*	2.05	1.98	2.04			
Question	17	17	17	0.63	0.73	
Low	49.30%	57.78%	46.75%			
Neutral	8.45%	11.11%	2.60%			
High	42.25%	31.11%	50.65%			
Mean	3.70	3.62	3.86			
*SD*	1.59	1.61	1.52			
Question	18	18	18	9.61	<0.01	**
Low	87.32%	64.44%	83.12%			
Neutral	0.00%	8.89%	2.60%			
High	12.68%	26.67%	14.29%			
Mean	2.34	3.07	2.23			
*SD*	1.39	1.60	1.56			
Question	19	19	19	4.1	0.13	
Low	76.06%	84.44%	71.43%			
Neutral	14.08%	15.56%	19.48%			
High	9.86%	0.00%	9.09%			
Mean	1.87	1.51	2.08			
*SD*	1.62	1.12	1.64			
Question	20	20	20	12	<0.01	**
Low	80.28%	57.78%	49.35%			
Neutral	0.00%	8.89%	15.58%			
High	19.72%	33.33%	35.06%			
Mean	2.58	3.44	3.47			
*SD*	1.50	1.63	1.83			
Question	21	21	21	13.76	<0.01	**
Low	70.42%	31.11%	48.05%			
Neutral	0.00%	28.89%	10.39%			
High	29.58%	40.00%	41.56%			
Mean	2.87	4.07	3.58			
*SD*	1.78	1.34	1.65			
Question	22	22	22	10.54	<0.01	**
Low	92.96%	77.78%	68.83%			
Neutral	0.00%	6.67%	14.29%			
High	7.04%	15.56%	16.88%			
Mean	1.66	2.22	2.49			
*SD*	1.19	1.54	1.70			
Question	23	23	23	9.19	0.01	*
Low	92.96%	75.56%	75.32%			
Neutral	0.00%	6.67%	5.19%			
High	7.04%	17.78%	19.48%			
Mean	1.54	2.16	2.30			
*SD*	1.17	1.68	1.80			
Question	24	24	24	0.46	0.79	
Low	32.39%	46.67%	37.66%			
Neutral	40.85%	11.11%	27.27%			
High	26.76%	42.22%	35.06%			
Mean	3.66	4.00	3.79			
*SD*	1.98	2.09	2.20			
Question	25	25	25	6.71	0.03	*
Low	78.87%	53.33%	67.53%			
Neutral	0.00%	2.22%	5.19%			
High	21.13%	44.44%	27.27%			
Mean	2.86	3.67	3.06			
*SD*	1.42	1.52	1.60			
Question	26	26	26	20.32	<0.01	**
Low	43.66%	26.67%	46.75%			
Neutral	38.03%	20.00%	27.27%			
High	18.31%	53.33%	25.97%			
Mean	3.14	4.91	3.39			
*SD*	1.91	1.74	2.07			
Question	27	27	27	5.55	0.06	
Low	46.48%	35.56%	48.05%			
Neutral	39.44%	26.67%	22.08%			
High	14.08%	37.78%	29.87%			
Mean	3.00	3.96	3.38			
*SD*	1.90	1.94	2.18			
Question	28	28	28	9.98	<0.01	**
Low	74.65%	42.22%	54.55%			
Neutral	1.41%	11.11%	10.39%			
High	23.94%	46.67%	35.06%			
Mean	2.87	3.84	3.48			
*SD*	1.52	1.69	1.67			
Question	29	29	29	2.67	0.26	
Low	74.65%	62.22%	54.55%			
Neutral	1.41%	6.67%	5.19%			
High	23.94%	31.11%	40.26%			
Mean	3.10	3.29	3.53			
*SD*	1.32	1.62	1.54			
Question	30	30	30	5.09	0.08	
Low	47.89%	31.11%	41.56%			
Neutral	2.82%	6.67%	12.99%			
High	49.30%	62.22%	45.45%			
Mean	3.86	4.44	3.96			
*SD*	1.42	1.63	1.55			
Question	31	31	31	13.48	<0.01	**
Low	45.07%	24.44%	40.26%			
Neutral	40.85%	24.44%	23.38%			
High	14.08%	51.11%	36.36%			
Mean	3.03	4.42	3.74			
*SD*	1.86	1.99	2.15			

*Note*. Summary from Likert analysis (left column) and Kruskal–Wallis rank sum test (right column).

a Never/seldom/N/A.

b Sometimes/fairly often.

c Very often/all the time.

* *P* value < 0.05.

** *P* value < 0.01.

### 3.1 Oral health questions, numbers 2, 3, 6, 8, and 16

In the OS group, 32.47% answered that during the last year, they had difficulties in chewing due to problems with the teeth, and 42.86% had problems with caries. They also had problems with altered taste (35.06%) and toothache (35.06%), and 48.05% had avoided eating some foods. These differences were statistically significant compared with the HNS and ONS groups (*P* < 0.01).

### 3.2 Oral health questions, numbers 7 and 9

In the ONS group, 42.22% stated that they have had significantly more difficulties with sensitive teeth and 35.56% with painful aching from the oral mucosa compared with the two other groups (*P* < 0.05).

### 3.3 Psychological well‐being and dental health questions, numbers 20, 21, and 22

In the OS group, 35.06% had been worried by dental problems, 41.56% had felt uncomfortable about the appearance, and 16.88% were more likely to have felt uncomfortable about the appearance regarding the teeth. The ONS group gave similar answers concerning psychological well‐being and dental health‐related questions as the OS group; 33.33% had been worried by dental problems, 40.00% had felt uncomfortable about the appearance, and 15.56% were more likely to have felt uncomfortable about the appearance regarding the teeth. These problems were prevailed in the OS and ONS groups compared with the HNS group (*P* < 0.01).

### 3.4 Psychological well‐being and dental health questions, numbers 23 and 25

In the ONS group, 44.44% also declared to have felt depressed (*P* < 0.05). Moreover, 19.48% of the OS group and 17.78% of the ONS group stated that they had avoided smiling, compared with the HNS group (*P* < 0.05).

### 3.5 General health and quality of life questions, numbers 26, 28, and 31

The ONS group also reported more problems with general health‐related questions as heartburn 26.67%, the feeling of worsened general health 53.33%, insomnia 46.67%, and difficulties in doing usual jobs 51.11% (*P* < 0.01).

## 4 DISCUSSION

The present study was undertaken to evaluate the perceived oral health amongst individuals that had undergone bariatric surgery and its possible impact on quality of life. Self‐reported oral health problems were more common in individuals that have undergone bariatric surgery than in obese patients and healthy people of normal weight. This may signify that individuals that have undergone bariatric surgery (OS group) are more likely to have oral health problems than others. The dietary recommendations for individuals that have undergone bariatric surgery vary from an intake of five to seven meals per day and to wait with intake of liquids approximately 30–60 min after having the meal or 30 min before (Jastrzebska‐Mierzynska et al., 2015). Recommendations given from dental health practitioners, on the other hand, are that patients are encouraged to reduce the frequency of intakes, and liquids should be ingested at mealtime in order to avoid prolonged acid attacks known to be one of the risk factors for dental caries (Moynihan, 2002). In our study, the OS group reported a relatively low positive response on the question “been eating more than 5 meals/snacks/liquids per day except from water” and a significant difference compared with the other two groups regarding problem with caries (*P* < 0.01). Thus, the theory of a higher number of intakes due to surgery and the connection with a higher prevalence of caries cannot be certified in our study. However, previous studies (Hague & Baechle, 2008; Marsicano, Grec, Belarmino, Ceneviva, & Peres, 2011) have demonstrated noticeably higher caries activity in patient with a history of bariatric surgery and also highlighted that the importance of postsurgical meal patterns may place the patient at an increased risk for dental caries, particularly in the presence of other risk factors. Another risk factor for caries is the constitution of the food and liquids regarding the content of carbohydrates, especially sugar (Zero, 2004). We did not ask which type of food and liquids the study participants used to eat and drink. This is a deficit of the study, and we can therefore only make an assumption that there could be a connection between a prolonged acid attack pattern and the meal content, which could explain why the OS group state a higher experience regarding caries, toothache, and difficult to chew because of problems with the teeth. The OS group reported an almost significant higher positive response regarding hyposalivation and intake of sweets more than once per day. Dehydration can be seen in the early postoperative period after all bariatric procedures (Shikora, Kim, & Tarnoff, 2007) and is mainly due to decreased fluid intake. Common side effects such as vomiting and/or diarrhea may exacerbate fluid losses. Hyposalivation together with gastric disorders such as nausea, heartburn, and vomiting, which are also commonly seen after bariatric surgery, are factors that may contribute to increased dental wear and hypersensitivity. According to Heling et al. (2006), 37% of patients who had undergone bariatric surgery experienced an increased problem with dental hypersensitivity. An interesting observation in our study was that the ONS group showed a higher prevalence of dental hypersensitivity than the OS group. However, the prevalence of dental hypersensitivity was higher in the OS group compared with the HNS group. This matches the results from our survey where the ONS group more often suffered from heartburn than the OS group and the HNS group. Surprisingly, our data showed that heartburn was a relatively uncommon problem in the OS group, as in the HNS group. None of the groups had an extensive problem with vomiting although this is a commonly reported side effect in the literature (Barbosa et al., 2009).

Oral hygiene is a basic factor to good oral health. When analyzing the oral hygiene results, no statistical significance could be found between the different groups. Previous studies investigating how bariatric surgery affects the periodontal status have reported contradictory results (Cummings & Pratt, 2015; de Moura‐Grec et al., 2014; Marsicano et al., 2011; Netto et al., 2012; Pataro et al., 2012; Sales‐Peres et al., 2015). According to Sales‐Peres et al. (2015), an increased systemic inflammation could be seen in patients after gastric bypass surgery, but this did not seem to affect the course of periodontal disease. However, the severity of periodontitis was increased after surgery, and a slightly increase of the periopathogenic bacteria, Porphyrmona gingivalis, could be detected. Moreover, different types of other periopathogenic bacterias detected decreased after surgery. De Moura‐Grec et al. (2014) found in their study an increase of periodontal disease 6 months after surgery compared with before surgery. Pataro et al. (2012) claimed in their study that even if the periodontal conditions altered, periodontitis was high in patients both before and after surgery. The question in our study, which could reveal signs of periodontal disease, showed no statistical significance in terms of bleeding gums between the groups, but the ONS and the OS groups showed a higher occurrence of bleeding gums than the HNS group. Pain from the oral mucosa can also be a sign of periodontal disease, and we found a statistic significance where the ONS group seem to have greater problems with painful gums/tongue/oral mucosa than the remaining groups. However, painful oral mucosa was twice as common in the OS group compared with the HNS group.

Lifestyle changes after bariatric surgery may develop or increase the severity of preexisting dental problems. However, results from present study showed that these alterations in oral health did not influence the quality of life. Individuals that have undergone bariatric surgery reported higher or similar quality of life compared with obese patients yet still lower quality compared with healthy people of normal weight. This replicates previous studies who also found that quality of life was significantly enhanced after bariatric surgery (de Zwaan et al., 2002; Kolotkin, Crosby, Gress, Hunt, & Adams, 2009; Major et al., 2015; Sarwer et al., 2010). In this study, the ONS group reported a higher positive response on questions regarding the feeling of worsened general health, insomnia, depression, and to have difficulties conducting usual jobs. The OS group reported less positive responses on those questions than the ONS group but higher positive response than the HNS group. Both the OS group and ONS group reported that they are more likely to have been worried by dental problems, are more likely to have felt uncomfortable about the appearance, and are more likely to have felt uncomfortable about the appearance of their teeth. Thus, we can draw the conclusion from the present study that the general feeling of well‐being is better after surgery in several ways but still lower than in the HNS group. It can be observed (Table 1) that the ONS group was the relatively oldest group and the group with the highest average BMI. An additional noteworthy observation was that the distribution of gender was skewed towards female patients, and the mean age in the HNS group was considerably lower compared with the two obese groups. These differences need to be considered when evaluating quality of life. Even though OHIP‐S (Larsson et al., 2004) is proven to have high reliability and to be a reliable measurement tool to examine the connection between oral health, psychological well‐being, and quality of life, we adopted a modified version, which may render the results less trustworthy. Additionally, the reliability of a survey as a measurement tool is limited and only reflects the study participants' subjective experience, which can differ between individuals. To obtain a more objective result, it would be desirable to register patient‐reported outcomes before eventual bariatric surgery and afterwards to rule out that the individuals who undergo this type of surgery are not different from the individuals who choose not to, in terms of oral health and oral health‐related quality of life. Even better would be to strengthen such investigations by establishing their oral health status through clinical examinations. It would be advantageous to have a more equal distribution of study participants in the three groups. The OS group seemed to be much more eager to partake in the present study. Our interpretation is that those individuals are perhaps more motivated to respond because they have undertaken a substantial change of lifestyle. Overweight is stigmatized in today's society, and our interpretation is that those individuals might not be too keen to be reminded of their situation and to be exposed as obese. The present study had a large proportion of female study participants. Admittedly, a potentially more reliable result could have obtained if only female study participants were included, because of differences in hormone level and body constitution. Interestingly, women seem to be overrepresented in the clinical studies referred to earlier in this paper. Although more research is needed, dental health‐care professionals should consider bariatric surgery as a potential risk marker for impaired oral health. Not all individuals that have undergone bariatric surgery develop oral health problems after surgery, therefore information and prophylaxis on an individual basis seems to be beneficial in order to prevent progression of oral and dental manifestations linked to bariatric surgery.

Future research, to investigate the oral microflora before and after surgery, clinical examinations with long follow‐up aiming to monitor the oral health development in bariatric surgery patients over time, and more detailed and specific surveys regarding oral health, dental hygiene habits, and visits to dentist, food consumption patterns, and dietary intake are needed to achieve a better understanding.

In conclusion and within the limitations of this case–control study, self‐reported oral health problems are more common in individuals that have undergone bariatric surgery than in obese patients and healthy people of normal weight. Individuals that have undergone bariatric surgery self‐reported a higher or similar quality of life compared with obese patients; thus, the quality was still lower compared with healthy people of normal weight. The findings of this study provide additional and valuable information following bariatric surgery and possible impact on oral health.

## CONFLICTS OF INTEREST

The authors report no potential conflicts of interest.
